# Supplementary material for: Factors Influencing Gaseous Emissions in Constructed Wetlands: A Meta-Analysis and Systematic Review
Source: Int J Environ Res Public Health. 2023 Feb 22;20(5):3876. doi: 10.3390/ijerph20053876 (PMC10001287; doi:10.3390/ijerph20053876)
Supplement: Supplementary file 1 [file ijerph-20-03876-s001.zip › ijerph-2184641-supplementary.pdf]

Figure S1. The flow diagram of literature search and selection process.

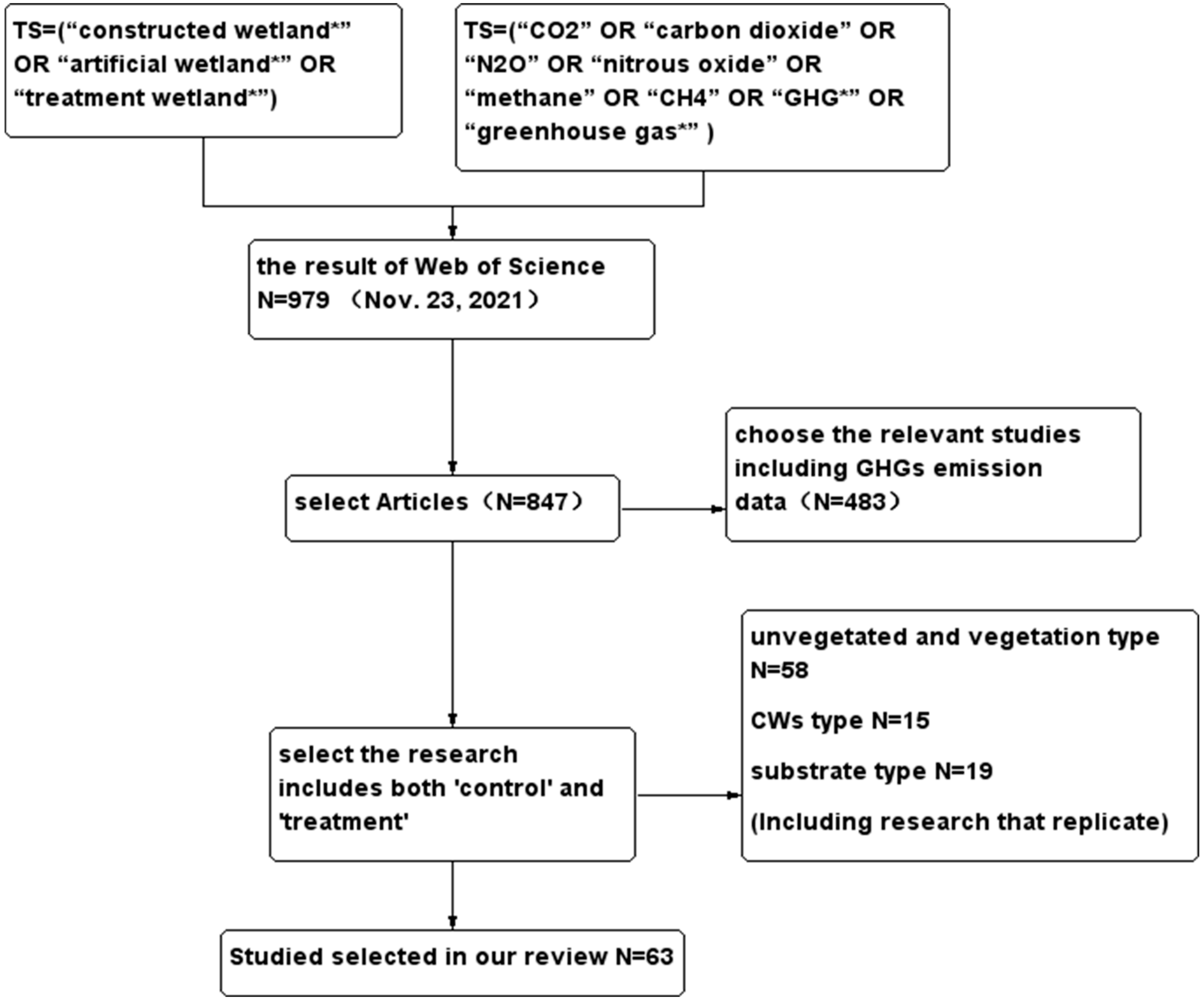

Table S1. CH_4 and N_2O emission from different substrate- amended CWs.

CWs type	Substrate type	Wastewater type	Vegetation	CH_4 flux ($mg/m^2/h$)	N_2O flux (($mg/m^2/h$))	Reference
FWS	No treatment	Agricultural runoff	*Holcus lanatus* grass	27.0		[37]
	Iron ochre	--	--	9.0		
	Gypsum	--	--	28.0		
VSSF	Gravel	Synthetic wastewater	*C. alternifolius*	229.2		[35]
	Manganese ore	--	--	125.4		
VSSF	Quartz sand, anaerobic sludge	Synthetic wastewater	*I. pseudacorus*	0.048 - 0.051		[36]
	Iron ore-amended	--	--	0.059 - 0.061		
VSSF	Gravel, quartz sand	Artificial wastewater	*I. pseudacorus*	0.06	0.003	[39]
	Iron ore-amended	--	--	0.05	0.004	
	Manganese ore-amended	--	--	0.00	0.003	
VSSF	Gravel, sand	Synthetic wastewater	*I. pseudacorus*	17.08	0.10	[38]
	Walnut shell-amended	--	--	252.30	0.07	
	Manganese ore-amended	--	--	2.00	0.14	
	Activated alumina-amended	--	--	6.43	0.20	
VSSF	Sand, gravel, coarse gravel	Synthetic wastewater	*I. pseudacorus*	0.154		[118]
	Biological ceramic-amended	--	--	0.157		
	Magnetite-amended	--	--	0.186		
	Sand, gravel, coarse gravel	Diluted swine wastewater	--	0.043		
	Biological ceramic-amended	--	--	0.105		
	Magnetite-amended	--	--	0.095		

Note: The symbol “--” indicates the same content as the line above.

Table S2. NH_3 volatilization from CWs and ponds is affected by several factors.

CWs type	Wastewater	TAN and/or TN (mg/L)	Plant	Temperature °C	pH	NH_3 (mg/m^2/d or % of TN)	Reference
FWS CWs	Swine	223			7.0	816.0	[100]
		117	*T. latifolia*		7.1	48.0	
		113			7.3	216.0	
		102			7.1	504.0	
Marsh	Swine	162		24.5	7.0	360.0	
Marsh		60		20.2	7.1	240.0	[12]
Pond		59	*T. latifolia*	26.9	7.4	1824.0	
Marsh		6		23.5	6.6	96.0	
Pond		5		26.4	6.9	120.0	
Marsh		4		26.5	6.6	48.0	
FWS CWs	Dairy	254				130.0	
		229	*T. latifolia*			205.0	[11]
		254				518.0	
		258				251.0	
SSF CWs		291				15.0	
		244				24.0	
		285				73.0	
		297				99.0	
FWS CWs	Swine	136.4	*T. angustifolia*	13.7	7.5	10.5% (of TN)	
		402.0		20	7.2	12.3% (of TN)	[101]
		549.9		16.9	7.3	20.4% (of TN)	

CW microcosm	Swine	213.5 (TN)	Without	22.7-35.8		224.2	[119]
			A. philoxeroides			77.8	
			M. aquaticum			69.9	
CW microcosm	Synthetic	336	One species	18.7		0.8	[105]
			Two species			0.7	
			Three species			0.2	
			Four species			0.47	
Floating CW microcosm	Synthetic	336	One species	18.7		1.1	[63]
			Two species			0.9	
			Three species			0.7	
			Four species			0.6	
HSSF bed	Municipal	63.6 (TN)	*T. latifolia* dominate		6.9	1.7% (of TN)	[99]
					8.4	5.9% (of TN)	
					10.5	23.1% (of TN)	
					12.3	15.1% (of TN)	

Note: SSF=subsurface flow (some data were extracted by GetData).
